# Conflict and Health: seven years of advancing science in humanitarian crises

**DOI:** 10.1186/1752-1505-8-7

**Published:** 2014-05-12

**Authors:** Ruwan Ratnayake, Olivier Degomme, Bayard Roberts, Paul Spiegel

**Affiliations:** 1 International Rescue Committee, 122 E 42nd Street, New York 10168, USA; 2 International Centre for Reproductive Health, Ghent University, De Pintelaan 185 UZP114, Ghent 9000, Belgium; 3 Faculty of Public Health and Policy, London School of Hygiene and Tropical Medicine, 15-17 Tavistock Place, London WC1H 9SH, UK; 4 Division of Programme Support and Management, United Nations High Commissioner for Refugees, Geneva, Switzerland

## 

*Conflict and Health* began in 2007 with an aim to provide a forum to document public health responses during and after conflict across the world. The journal has published over 120 articles that span the range of public health domains including, but not limited to, infectious disease control, reproductive health and sexual and gender-based violence, mental health, health system reconstruction, and ethics in emergencies. The growth of *Conflict and Health* has taken place during a time of increasing focus on evidence-based approaches to reducing mortality and morbidity in humanitarian emergencies, and the increasing prominence of open-access peer-reviewed literature [1-3].

In a world that remains affected by armed conflict, the aim of the journal remains more relevant than ever. In 2014, conflict is ongoing in countries and regions as dispte as Central African Republic, Syria, South Sudan, Iraq, Myanmar, and the Sahel Region and Northern Nigeria. There is increasing awareness, action and data behind the idea that internally displaced people, refugees and increasingly, residents of countries, are affected by conflict [4].

Syria in particular provides a clear example of the all-encompassing nature of conflict on national health and the effects on regional development. Three years into the war, the scale of the humanitarian emergency in Syria and its neighbors is unprecedented. Most Syrians are at-risk, with an unknown current number of conflict-affected residents (nearly 12 million in 2012), 6.5 million internally displaced persons (IDPs) and nearly three million refugees spread over Egypt, Iraq, Jordan, Lebanon and Turkey in 2013 (and four million refugees projected by the end of 2014) [5,6]. The crisis has reversed Syria’s two decades of progress. In 2007, the proportion of the population living in extreme poverty was 0.3%. By 2012–2013, it is 7.2%, back to 1997 levels [7]. By mid 2013, the 70,000 documented deaths constituted a 50% increase in crude deaths during the pre-war period [8]. Almost two thirds of public hospitals are damaged or have no capacity. Measles vaccination coverage among one year olds dropped from 81% nationally in 2007 to 61% nationally in 2012 [9]. Similarly, oral polio vaccination coverage (OPV 3) among children under five dropped from 91% in 2010 to 68% in 2012, resulting in a poliomyelitis outbreak that emerged in Deir Ez Zur province in eastern Syria and Aleppo and spread to Iraq [10]. Nearly three million children have been vaccinated during a logistically challenging six rounds of massive vaccination from December 2013 to April 2014 [11]. Conversely, hypertension, diabetes and mental health issues make up the burden of disease and issues such as adherence to medications and continuum of care are essential. Cancer and kidney failure needing renal dialysis are also serious and expensive illnesses that have become prominent. Syrian refugees have fled to urban areas in Iraq, Lebanon, Turkey, Egypt and Jordan presenting multiple new challenges that the humanitarian field is not well-accustomed to sufficiently addressing, including urban refugees and the control of noncommunicable diseases.

Syria reflects the emerging challenges to humanitarian assistance. In the last two decades, the numbers of IDPs living within national borders continues to increase as compared to a fairly stable global number of refugees until the recent Syrian crisis [12,13]. In addition, conflicts have increased in middle-income countries in the Balkans, the Caucasus, and the Middle East, who have older populations compared to low-income counties and suffer proportionally more from diabetes, hypertension and other noncommunicable diseases, as opposed to the malnutrition and epidemic infectious diseases experienced in high-density camps in resource poor settings. Writing in *The Lancet*, Spiegel and others [3] highlighted key challenges and opportunities to address health in current conflict settings including: (1) rolling out health services via mass campaigns of new and underused interventions for maternal and neonatal health, airborne diseases and neglected tropical diseases to populations normally considered inaccessible; (2) systematically addressing chronic diseases including HIV/AIDS, tuberculosis, diabetes and cardiovascular disease; (3) planning and improving health services for conflict-affected populations in urban areas within existing health systems such as IDP populations in Nairobi, Cairo and Peshawar; and (4) improving and validating surveillance and monitoring of health status and population estimation of displaced people.

## The state of research

Developing more effective and flexible approaches necessitates research done with rigor. Yet, there are copious reasons for academics to avoid this enterprise altogether. Research in these settings is logistically difficult and there is limited funding. It is difficult to position research as a life-saving effort in the short-term. In the past, research has been cited as distraction to core medical, water and sanitation, nutrition and protection priorities and at times, research has been conducted in ethically problematic ways [14].

However, there is a strong need for the development of a basic understanding of the effectiveness, cost-effectiveness and delivery of interventions. At the broader level of the health system, how can services, systems and policies perform better and be more responsive to peoples’ needs? This is underscored by a recent review of health studies in humanitarian settings [15]. The review found a lack of studies that evaluated the delivery of interventions, particularly for those topics (i.e., infectious disease control) where evidence for the effectiveness of an intervention (i.e., the measles vaccine) exists. Other topics also lacked a body of research on effectiveness of specific interventions (i.e., gender-based violence and mental and psychosocial health). There was a lack of research among urban populations and mid to high income settings. Finally, health assessment methods and cost-effectiveness methods need attention. More positively, of the 706 studies reviewed, 76% were conducted in the last decade, showing an increased willingness to conduct research in humanitarian settings.

*Conflict and Health* provides a forum for demonstrating that rigorous research can be done in conflict-affected settings by academics and more crucially, local and nongovernmental organizations. It is clear that a greater commitment to research by academics, NGOs and governments is needed, but research donors play an important enabling role. Fortunately, the situation is changing as the recent R2HC programme provides a fund from DfID and the Wellcome Trust that was created with the explicit aim of building the evidence base for humanitarian intervention in complex humanitarian emergencies (see, http://www.elrha.org/work/r2hc). Health scientists from conflict-affected countries, who likely understand the context and should be contributing to research as disasters happen, should be better placed to navigate these systems [16,17].

Many field practitioners explicitly aim to fill the gaps that traditional academic researchers rarely explore, making important contributions to *Conflict and Health* and demonstrating innovation. MSF used a series of case studies to examine the successful provision of antiretroviral treatment (ART) during violent periods in a set of 22 countries where MSF was working [18,19]. An interagency group evaluated family planning knowledge and behaviors and access to family planning interventions across six conflict-affected contexts in Africa [20]. Even the most recently published article to date reported on a rarely explored area: the outcomes of an orthopedic rehabilitation program implemented after the final siege in Northern Sri Lanka [21].

*Conflict and Health* has brought particular attention to several under-researched areas (Table 1). Infectious disease control in emergencies still dominates (26%), which represents the predominance of the infectious disease burden among affected populations in Africa and Asia as well as the shift towards including HIV/AIDS programming for conflict-affected populations [19,22,23]. In addition to the review by MSF listed above, other practitioners examined the implementation of programs for provision of ART, adherence to ART and the challenges therein among conflict-affected populations in Kenya and Republic of Congo [18,24-26]. Mendelsohn and colleagues [24] conducted a systematic review of 17 studies of adherence to ART that found a range of adherence from 87 to 99.5%. Bellos and others [25] systematically reviewed the literature to define the burden of acute respiratory infection in crisis-affected populations, a first step in defining the evidence base for improved intervention. Of note, while studies of malaria, pneumonia and diarrheal diseases exist, Human African Trypanosomiasis, which is characteristic of Sub-Saharan Africa and which persists in conflict settings, has been the focus on both a review of its prevalence and a debate on its control [26,27].

**Table 1:** Publications in Conflict and Health by theme, 2007-2014

**Area of focus**	**Proportion**
Infectious diseases and HIV/AIDS	26%
Mental health	20%
Sexual and gender based violence	10%
Mortality	9%
Reproductive health	6%
Health systems	4%
Environmental health	3%
Humanitarian assistance	3%
Substance abuse	3%
Surgery	2%
Editorials	2%
Education	2%
Ethics	2%
Human Rights	2%
Injuries	2%
Noncommunicable disease	2%
Nutrition	2%
Social determinants of health	2%
Child health	1%

The second largest proportion of articles concern mental health (20%) and sexual and gender based violence (10%). Specifically, surveys of mental health symptoms among conflict-affected populations have been reported on from a wide variety of settings including Sub-Saharan Africa, India, Peru, Chechnya and Kosovo [28-32]. In contrast, nearly half of the sexual and gender based violence studies are from Eastern Democratic Republic of Congo which is notable, but likely emphasizes that research is not yet addressing the breadth of the problem across settings [33-37]. One systematic review looked at the confluence of the two areas by reviewing the effectiveness of mental health interventions for persons affected by sexual and gender based violence [38].

Mortality is a key indicator of the magnitude of the humanitarian situation and effectiveness of the humanitarian responses, and nearly 10% of the journal’s output has been reports of mortality using retrospective surveys, surveillance methods and meta-analyses, including those from Chad, Democratic Republic of Congo, Haiti, Iraq and Somali refugees in Kenya [39-46]. Reports on the performance, validity and evaluation of surveillance systems for mortality and morbidity in emergencies are lacking, although this is routinely done by humanitarian organizations. Reports on the performance, validity and evaluation of surveillance systems, survey methods and data collection methods in general in emergencies are lacking are lacking, and papers published elsewhere have highlighted the need for further work on these issues [47-50].

Other key areas have attained a niche within the journal. The backbone for ethical conduct for research in conflict settings was laid out by a team of academics and field practitioners from Médecins Sans Frontières (MSF) in 2009 [14]. Four observational studies assessed the associations between exposures to environmental contaminants resulting from war including mustard gas, uranium and nuclear pathogens in the Middle East and birth defects and morbidity [51-54]. Some of the rare reports on substance abuse including tobacco and alcohol among conflict-affected populations in Kenya, Liberia, Northern Uganda, Iran, Pakistan, and Thailand are found in the journal [55-58]. Similarly, four observational studies of surgery and occupational health following trauma have set the stage for further investigation of these important topics [21,59-61].

Only two articles were published on NCDs and it is clear that further research is required on NCDs given their rise globally, with NCD control as a major challenge in Syria and previous conflicts such as in the Balkans, the Caucasus and Sri Lanka [62]. The five articles published on health systems reconstruction scratch the surface of a largely neglected area. In particular, one of the articles takes a comprehensive view through the use of a case study framework to analyze the impact of the success of health sector reforms during the health sector reconstruction period in Kosovo [63]. Comprehensive studies of health sector reform in general are rarely found even elsewhere, but in general are now needed to document and inform current debates [64-66].

## Publication statistics

**Figure 1:**
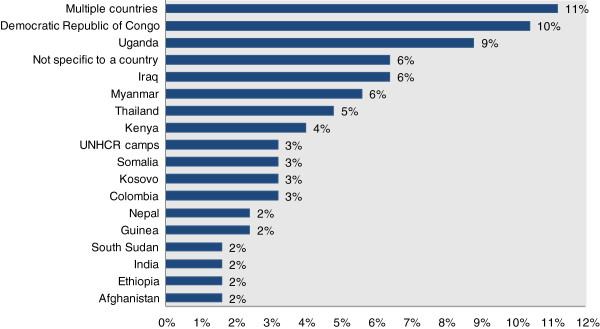
**Publications by country of study, for countries with greater than one article 2007–2014.** *Countries with less than 1% (or one article) include Armenia, Bangladesh, Bosnia and Herzegovina, Central African Republic, Chad, Chechnya, Haiti, Indonesia, Iran, Israel, Israel-Palestine, Liberia, Mozambique, Palestine, Peru, Philippines, Republic of Congo, Rwanda, Sierra Leone, Sri Lanka, Syria, Tanzania and Timor-Leste. Four articles from Canada and Sweden were excluded.

In 2013, the journal reached 194,000 website hits and more than 10,000 individual accesses of each of the top 10 accessed articles since the start of the journal. The full range of countries referred to in the journal represents those affected by conflict and natural disasters since the 1990s. In particular, five or more articles come from Democratic Republic of Congo, Uganda, Iraq, Thailand, Kenya and Myanmar. Countries that are considered forgotten conflicts including Somalia and Central African Republic also factor into the list. Nonetheless, papers from current and former conflicts in South America, West Africa and South Asia are largely absent (Figure 1).

**Figure 2:**
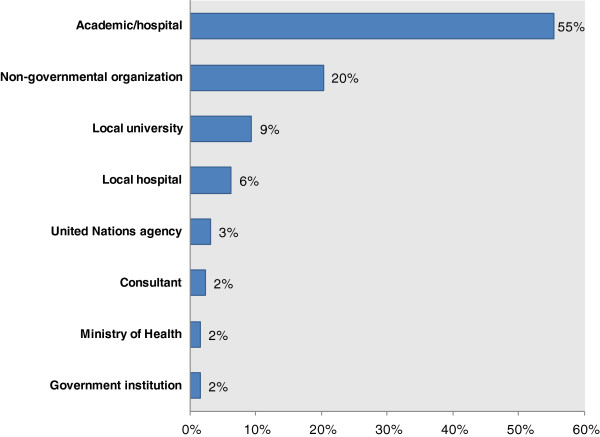
Publications by provenance of the first author, 2007–2014.

Analysis of *Conflict and Health* first authors reveals that research is still largely led by academics (55%) (Figure 2). However, non-governmental organizations such as MSF/Epicentre and Healthnet-TPO contribute a fifth of the articles (20%). Local universities and hospitals such as the Hawler Medical University in Iraq, the Rehabilitation and Research Centre for Torture Victims of Kosovo and Université de Goma in the DRC, make up 15% of the articles. McKee, Basu and Stuckler [67] conducted a useful analysis of the number of health research publications in peer-reviewed journals by national population size that puts this accomplishment into context. They found that DRC, Myanmar, Sierra Leone and nearly all of the other countries whose local hospitals and universities produced first authors for *Conflict and Health* have 0.13 to 1.5 health research publications per 100,000 population These are the lowest rates in the world, which McKee hypothesize is owing to poor research capacity, low political commitment to research and lack of infrastructure.

## The way forward

Armed conflict remains a serious threat to health that is currently destroying health systems and future capacity in places as dispte as Syria, the Central African Republic and Myanmar. It consistently undermines the Millennium Development Goals, efforts to implement universal health coverage and the basic human right to health. Seven years of the journal development has revealed promising opportunities. First, we are three new Editors-in-Chief and have added a new Editorial Board that explicitly integrates academics and technical experts from nongovernmental organizations including Epicentre, ICRC, International Rescue Committee and Médecins Sans Frontières and from affected countries. Second, though 15% of the articles are already led by authors from conflict-affected countries, efforts to strengthen the possibilities for authors from affected countries to contribute to the journal are warranted. At current, publication fees are waived for authors from low-income and low-middle income countries (see, http://www.biomedcentral.com/authors/oawaiverfund/). Other strategies may include engaging technical experts from affected countries to contribute reviews of topics of under-researched areas, and reports on their ongoing research studies and sponsoring writing workshops to ensure that the publication of high-quality, sound research is not stymied by a lack of experience in writing journal papers. Third, there is clear value in ensuring the journal has systems in place to rapidly evaluate and publish quality research in current crises (e.g. Syria, South Sudan, Northern Nigeria, Myanmar/Rakhine State) as well as less documented, forgotten crises (Sri Lanka, Myanmar/Kachin crisis, Columbia, Yemen) to have more immediate benefits on practice. Fourth, we will address the dearth of evidence for challenging areas including the delivery methods for health care, epidemiology and control of noncommunicable diseases, integration of displaced populations into health systems and monitoring and surveillance of displaced populations and areas that require elucidation of the evidence base [3,15]. To this end, the journal is collaborating on two special issues on the global evaluation of the work of the Interagency Working Group on Reproductive Health in Crises (IWAG), and health systems in fragile and conflict affected states in partnership with Health Systems Global and the Global Symposium on Health Systems Research (see, http://blogs.biomedcentral.com/bmcblog/2014/02/18/call-for-papers-filling-the-void-health-systems-in-fragile-and-conflict-affected-states/), and further special issues are planned. Last, given the increasing volume of publications and the need to motivate academics to publish with the journal, we intend to apply for an Impact Factor.

The points of action mentioned above will be crucial to ensure a sustained growth of the journal, but to ensure that scientific evidence guides public health interventions in humanitarian settings, we will have to go one step further. In order for *Conflict and Health* to contribute to improving the lives of conflict-affected individuals, we will ensure that findings presented in the journal are easily understood and of direct relevance to policy-makers, and we will strive for a high uptake of the key findings published in our articles.

Some practitioners once called the lack of generation of feasible solutions for the treatment of AIDS among conflict-affected populations a “failure of the imagination” [68]. Case studies in *Conflict and Health* and other journals have shown progress in ART treatment during conflict. Similarly, we hope that the next ten years of *Conflict and Health* will encourage public health practitioners and researchers in the North and South alike to push their creative ideas to develop rigorous research and sound publications that will fill the void, push the field forward and save and improve lives.
